# Metabolomics Reveal Key Metabolic Pathway Responses to Anxiety State Regulated by Serotonin in *Portunus trituberculatus*

**DOI:** 10.3390/metabo14100568

**Published:** 2024-10-21

**Authors:** Wei Zhai, Yuanyuan Fu, Lei Liu, Xinlian Huang, Sixiang Wang

**Affiliations:** 1 School of Marine Sciences, Ningbo University, Ningbo 315211, China; zzw18758304182@163.com (W.Z.); huangxinlian1997@163.com (X.H.); sixiangwang2021@163.com (S.W.); 2 Ningbo Institute of Oceanography, Ningbo 315832, China; fuyuan0101@163.com

**Keywords:** metabolomics, *Portunus trituberculatus*, serotonin, anxiety, aggressive behavior, clonazepam, OFT, crustacean

## Abstract

Background: Anxiety refers to the pathological persistence and intensification of emotional responses to danger, affecting health from psychological and physical aspects. Serotonin is an important neurotransmitter involved in the onset of anxiety. Methods and Results: To explore the biological changes in the formation of anxiety in crustaceans under the regulation of serotonin, we applied the open field-like test method for assessing anxiety states of larval *Portunus trituberculatus*, a highly aggressive crustacean species with a more simple neural structure compared with rodents and mammals. Compared with the control group, serotonin treatment resulted in a significant decrease in the time spent by the larvae in the central zone, suggesting anxiety-like behavior. Clonazepam treatment reversed this result and provided further evidence that the behavior of larval *P. trituberculatus* displayed anxiety. Moreover, a non-targeted metabolomic analysis found a significant alteration in the metabolites involved in tryptophan metabolism pathways associated with anxiety, including L-kynurenine, N-acetyl serotonin, and serotonin. These metabolites are involved in the serotonin pathway, the kynurenine pathway, and other pathways that affect anxiety through tryptophan metabolism. There were no significant differences in tryptophan metabolism levels between the control and clonazepam treatment groups. Conclusions: Our results demonstrate the possible existence of anxiety-like behavior in the larvae of *P. trituberculatus* from two perspectives. Being a species with a simpler neural structure than that of mammals, the larvae of *P. trituberculatus* offer a convenient model for studying the mechanisms of anxiety in crustaceans.

## 1. Introduction

Anxiety is a subjective sensation of tension and restlessness produced by external or internal pressures [1], and anxiety disorder is one of the most prevalent psychiatric conditions worldwide [2]. Approximately 264 million people suffer from anxiety disorders [3]. Anxiety is often accompanied by the development of depression, a condition that can have serious consequences, such as insomnia [4], cognitive impairment [5], and even suicide [6]. Therefore, it is necessary to explore the biological mechanisms of anxiety to develop effective treatments and drugs. Rodents, being highly similar to humans in physiology, anatomy, and genetics, have become the best experimental models for studying the effects of anxiety on health and behavior. For example, anxiety can lead to shorter life spans in adult mice [7], and the more similar the anxiety level, the easier it is for mice to mate and breed [8]. Previous studies have shown that anxiety can alter animal aggressive behavior [9,10], exploratory behavior [11], defense behavior [12], and social and cognitive behaviors [13,14,15]. In recent years, researchers have investigated the mechanisms underlying psychiatric conditions by exploring and evaluating the effect of anxiety on animals using methods such as the elevated plus maze (rats) [16], the submerged plus maze (fish) [17], the open field-like test (OFT-like) [18], light/dark boxes [19], the Morris water maze [20], and the forced swim test [13].

The swimming crab (*Portunus trituberculatus*) is an economically important commercial fishery species with the advantages of rapid growth and high nutritional value, with proteins, minerals, and other nutrients in the meat [21,22]. The species is widely distributed in China, Japan, and other East Asian coastal countries [23]. In China, it is one of the main farmed crustaceans, with 105,283 tons produced in 2022. However, with the expansion of the market and the increase in demand, the larvae of the swimming crab are currently in short supply [24]. At the same time, swimming crabs have an extremely low survival rate in pond culture, with only 11% surviving the larval stages and only 10% reaching the adult stage [24,25]. Aggressive behavior and cannibalism are the major reasons for the extremely low survival rate, behaviors that are associated with food resources [26], living space [27], and territories [28]. Previous studies have indicated that anxiety can affect aggressive behavior in mammals [29,30,31], which are similar to humans in their nervous system, behavior reaction, and other factors. Crustaceans have a simpler nervous system than mammals. However, a previous study on *Procambarus clarkii* demonstrated that crustaceans also display anxiety-like behavior [32]. The mechanisms behind the formation of anxious behaviors in crustaceans have rarely been studied and thus are currently unclear. Therefore, it is necessary to explore the mechanism of anxiety-like behavior in swimming crabs.

Serotonin is a neurotransmitter that plays a significant role in regulating cognitive and behavioral functions, including feeding [33], aggression [25], sleep [34], anxiety [35], and memory [36]. Previous studies have confirmed the role of serotonin in anxiety, but there is still debate concerning its specific effects. It is generally believed that an increase in anxiety is associated with decreased serotonin levels [37,38,39], but increasing studies suggest that increased serotonin levels may also lead to heightened anxiety [40]. This has been well-demonstrated by the application of selective serotonin reuptake inhibitors (SSRIs). Over the past few decades, SSRIs, as common anti-anxiety medications, have been shown to alleviate anxiety by blocking the neuronal reuptake of 5-hydroxy tryptophan (serotonin) and thereby increasing serotonin levels [41,42,43]. However, acute treatment with SSRIs in some animal models, such as rats, has been found to increase anxiety-related behaviors [44]. This has also been observed in the eastern mosquitofish, in which SSRIs alter anxiety behaviors, with varying outcomes influenced by sex and concentration [45]. The regulation of anxiety by serotonin is complex, potentially involving individual genetics, receptor types, and neural networks. Further exploration into serotonin’s effect on anxiety is needed.

Metabolomics is a tool employed to comprehensively examine biological systems and the metabolic processes within cells, tissues, or organisms and finally obtain useful information in sampling [46,47]. Metabolomics combines metabolite profiling and multivariate data analysis to complete the high-throughput analysis of small molecular metabolites in biological samples [48]. The method was developed as an excellent molecular biological tool and has found widespread application in many areas of bioscience, including nutrition and medicine [49], biomarker discovery [50], environmental analysis and systems biology [51], and toxicology [52]. Currently, metabolomics is used to investigate the metabolic characteristics and affected pathways in mice under different levels of anxiety, thereby providing new insights for study and treatment [53,54]. Moreover, in human studies, approximately 17 age-related metabolites have been confirmed to be associated with anxiety [55].

In this study, we first used the OFT-like to observe anxiety-like behavior in swimming crabs and applied liquid chromatography–mass spectroscopy (LC-MS) to examine the contents of metabolites in different treatment groups. Multivariate analysis was employed to identify differential metabolites and metabolic pathways. Understanding the metabolomic underpinnings of anxious behavior in swimming crabs will deepen our understanding of marine invertebrate neurobiology and may have broad implications for commercial aquaculture.

## 2. Materials and Methods

### 2.1. Animals

Individuals of the first zoea stage of *P. trituberculatus* (Z1) were obtained from a cultivation farm in Ningbo, Zhejiang, China. A total of 500 zoea with intact limbs and active behavior were collected. The zoea of *P. trituberculatus* were temporarily raised in buckets, maintaining a temperature of 25 °C and a salinity of 20‰. Oxygen pumps were used for continuous aeration, and a light/dark cycle of 12 h was maintained. Baskets were placed in the buckets as shelters to reduce the effect of mutual aggression.

### 2.2. Drug Treatments

Drugs were administered to the zoea of *P. trituberculatus* through immersion. Firstly, we conducted more than three preliminary experiments before the experiment by exploring the effects of serotonin and clonazepam on the behavior of *P. trituberculatus* at stepped concentrations. Three gradient concentrations of 5-HT were set at 5 × 10^−3^ mol/L, 5 × 10^−5^ mol/L, and 5 × 10^−7^ mol/L to carry out the experiment, and the appropriate experimental concentration of 5 × 10^−3^ mol/L that did not affect the survival rate of the experimental subjects was selected. Then, serotonin with a concentration of 5 × 10^−3^ mol/L was used for 30 min of immersion to ensure absorption and efficacy In order to confirm that the behaviors affected by serotonin were anxious behaviors, clonazepam, an anti-anxiety drug, was selected for verification. Clonazepam with a concentration of 0.125 mg/L was applied for another 30 min after serotonin immersion using a fresh solution, which had no effect on the behavior and survival rate of *P. trituberculatus*. For all experiments, at least 24 replicates were set up in each group.

### 2.3. Open Field-like Test

**Figure 1:**
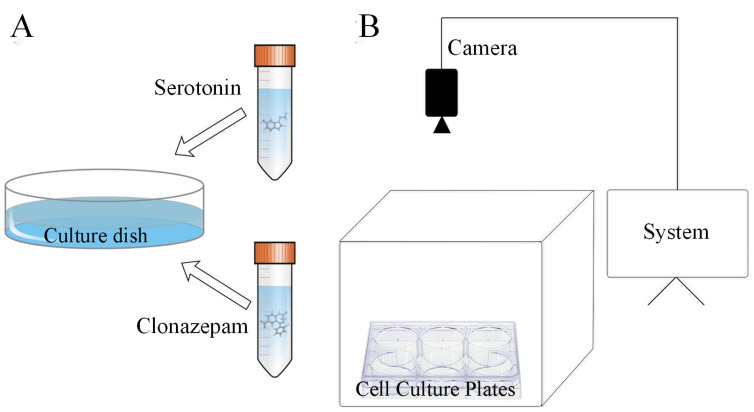
Schematic of materials and equipment used in the experiment. Section (**A**) shows materials for the drug treatment of larval *P. trituberculatus*. Section (**B**) is a brief schematic of the behavior tracking system. Serotonin and clonazepam were dissolved and poured into different culture dishes (diameter = 100 mm). The larvae were placed in the culture dishes, immersed in the drug solutions for 30 min, and then passed into the system. The system contained six-well cell culture plates and six targets that could be monitored at the same time. Behavioral recording lasted for 30 min, and the data were analyzed using computer programs (EthoVision XT 12.0, Noldus, The Netherlands).

The open field-like test (OFT-like) is an experimental method to judge anxiety by observing the residence time and movement status of the subjects in the central and marginal areas. Before the experiment, the zoea of *P. trituberculatus* were treated with the relevant drugs and then placed in a six-well plate (diameter = 3.6 cm) set within a zebrafish behavioral tracking system (DanioVision, Noldus, The Netherlands). The crabs were allowed to freely explore for 30 min. Then, video tracking software (EthoVision XT 12.0, Noldus, The Netherlands) was used to record and analyze the movement distances of the crab zoea and time spent in the central area to assess the influence of the drugs on anxiety-like behaviors (Figure 1). A shorter duration in the central area indicated stronger anxiety-like behavior.

### 2.4. Liquid Chromatography-Mass Spectrometry Analysis

A total of 216 larvae were randomly selected and divided into three groups, including the control group, 5 × 10^−3^ mol/L serotonin group, and 0.125 mg/L clonazepam-treated group for liquid chromatography–mass spectrometry analysis. Each group consisted of 6 replicates, and 12 individuals in each sample. The instruments used in LC-MS analysis included electronic balances (FA2104B, YuePing Ltd., Shanghai, China), whirlpool oscillators (TYXH-I, LanYi Ltd., Shanghai, China), automatic sample rapid grinding machines (Wonbio-E, Shanghai, China), ultrasonic cleaners (F-060SD, FuYang, Shenzhen, China), benchtop high-speed refrigerated centrifuges (TGL-16MS, LuXiang, Shenzhen, China), freeze-concentration centrifugal dryers (LNG-T98, Beijing, China), high-resolution mass spectrometers (QE plus, ThermoFisher, Shanghai, China), high-performance liquid chromatography (ACQUITY UPLC I-Class plus, Waters, MA, USA), and chromatographic columns (ACQUITY UPLC HSS T3 (100 mm × 2.1 mm, 1.8 μm),Waters, MA, USA).

The reagents used in the experiment included methanol (ThermoFisher, Shanghai, China, HPLC, 99.9%), acetonitrile (fisher, HPLC, 99.95%), formic acid (ThermoFisher, Shanghai, China, HPLC, 99.0%), water, and L-2-chlorophenylalanine (Shanghai HC biotech, Shanghai, China, HPLC, 98.0%).

The whole body of *P. trituberculatus* zoea was sampled with each weighing 20 mg, which was placed into a 1.5 mL Eppendorf tube, and methanol–water (methanol:water = 4:1, L-2-chlorophenylalanine 4 μg/mL) was added. After pre-cooling, the sample was sonicated (60 Hz, 2 min) in an ice–water bath for 10 min and incubated at −40 °C for 30 min. The samples were then centrifuged at 12,000 rpm for 10 min (4 °C); the supernatant was collected; 300 μL of methanol–water was added; and the sample was vortexed for 30 s. This was followed by ice–water bath ultrasonication for an additional 3 min, and the samples were finally incubated at −40 °C for 2 h before centrifugation (12,000 rpm, 4 °C, 10 min). The supernatant was collected and stored at −80 °C. Chromatography was performed using an ACQUITY UPLC HSS T3 column (100 mm × 2.1 mm, 1.8 μm) maintained at a temperature of 45 °C. The mobile phase consisted of A, water (with 0.1% formic acid), and B, acetonitrile. The flow rate was set at 0.35 mL/min, with an injection volume of 3 μL. All the samples were maintained at 4 °C during the analysis. The mass range was from *m*/*z* 125 to 1000. The resolution was set at 70,000 for the full MS scans and 17,500 for HCD MS/MS scans. The Collision energy was set at 10, 20, and 40 eV. The mass spectrometer operated as follows: spray voltage, 3500 V (+) and 3500 V (−); sheath gas flow rate, 40 arbitrary units (+) and 35 arbitrary units (−); auxiliary gas flow rate, 10 arbitrary units (+) and 8 arbitrary units (−); and capillary temperature, 320 °C.

### 2.5. Statistical Analysis

Experimental data were analyzed using SPSS 26.0 software, with one-way ANOVA used to compare differences between groups. After ANOVA, we carried out post hoc tests, including Duncan and Tukey’s. A *p*-value of less than 0.05 was considered to indicate a statistically significant difference, while a *p*-value of less than 0.01 denoted a highly significant difference. GraphPad Prism 8.0 was used for graphical representation. Xcalibur software Version 4.2 was employed for analyzing raw metabolomics data and generating total ion current chromatograms. The Simca program and R programming language were used to draw the OPLS-DA figures and others.

### 2.6. Ethical Note

All experiments were conducted following the recommendations in the Guidelines for the Laboratory animal-Guideline for ethical review of animal welfare.

## 3. Results

### 3.1. Anxiety-like Behavior Assays

**Figure 2:**
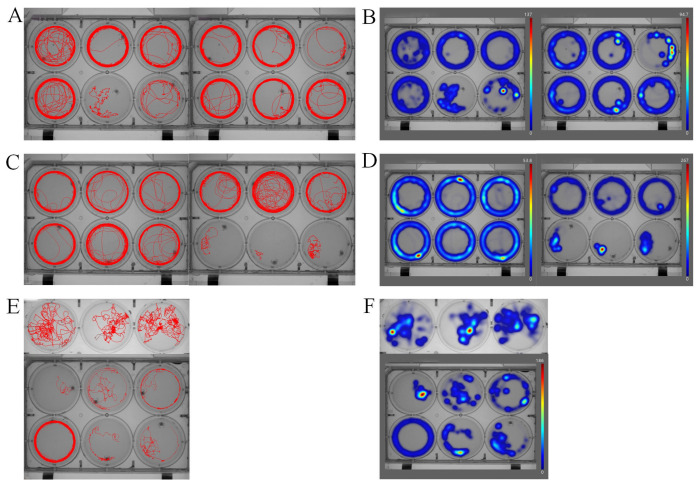
The motion trajectory maps and heat map diagram for the analysis of movement of *P. trituberculatus* larvae recorded by the behavior trajectory tracking system. The motion trajectory maps (**A**) and heat maps (**B**) of the control group. The motion trajectory maps (**C**) and heat maps (**D**) of *P. trituberculatus* larvae after serotonin treatment. The heat maps (**F**) and trajectory maps (**E**) following clonazepam treatment demonstrated the elimination of serotonin-induced anxiety-like behavior. The larger the value represented by the color in (**B**–**D**), the more frequent the crab’s movement frequency at a certain location.

**Figure 3:**
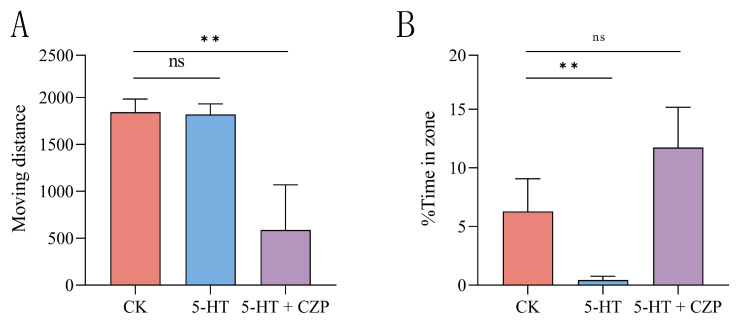
Analysis of locomotor activity and anxiety state of the larvae of *P. trituberculatus*. (**A**) The analysis of the total movement distance between the control group and the serotonin group. The analysis of locomotor activity between the 5-HT + clonazepam-treated group and the control group, and they exhibited a significant difference. (**B**) The analysis of the proportion of time spent in the central zone between the control group and the serotonin group. The 5-HT group exhibited a significant difference with the CK group. The analysis of the proportion of time spent in the central zone between the clonazepam-treated group and the control group. The 5-HT + CZP group exhibited no significant difference from the CK group. Note: ns indicates no significant differences between groups; ** indicates significant differences between groups (*p* < 0.01).

Before the experiment, in order to determine the most appropriate concentrations of 5-HT and CZP, we referred to a large number of studies and conducted a large number of pre-experiments. Multiple dilution of CZP, from 2 mg/L to 0.125 mg/L, was finally found to have a significant effect on the active substance of the experimental subjects. Finally, the drug concentration group with the best activity of the experimental subjects was selected (0.125 mg/L). We evaluated anxiety-like behavior in *P. trituberculatus* using the OFT-like. The crabs in the serotonin (5 × 10^−3^ mol/L) group showed significant reductions in the time spent in the central area and the ratio of time remaining in the central area compared with the control group (*p* < 0.01) (Figure 2). However, this phenomenon could be altered by the administration of clonazepam. When treated with clonazepam, the time spent in the central area and the proportion of time remaining in the central area in the OFT-like significantly increased (*p* < 0.01) (Figure 3). The results showed that, compared with the control group, the central area residence time of the serotonin-treated *P. trituberculatus* was significantly decreased, indicating an anxiety-like behavior after serotonin treatment. The behavior pattern of *P. trituberculatus* returned to the same level as that of the control group after treatment with the anti-anxiety drug clonazepam that apparently eliminated the anxiety-like state.

### 3.2. Metabolomic Multivariate Analysis of Serotonin and Clonazepam Treatment

**Figure A1:**
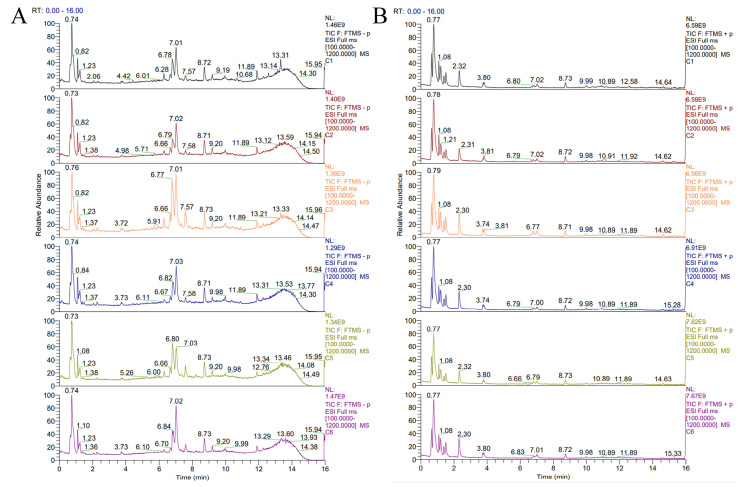
Total ion chromatography results for the control group under positive and negative ion modes. (**A**) Positive ion mode. (**B**) Negative ion mode.

Before the analysis of the results, we tested the change in total ionic strength with time and ensured the stability and accuracy of the data (Appendix A, Figure A1).

### 3.3. Principal Component Analysis Model

**Figure 4:**
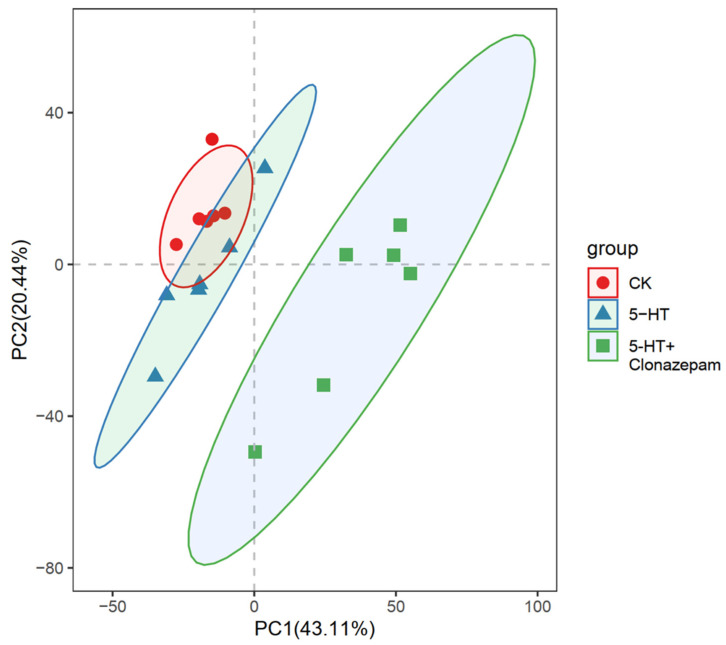
Principal component analysis of the CK, 5-HT, and clonazepam groups.

To investigate the internal biological changes in the zoea of *P. trituberculatus* under different treatments, untargeted metabolomics analysis was conducted on 18 samples to detect variations in metabolites. We performed PCA analysis on the content of 5515 metabolites detected in the samples. Principal component analysis (PCA) revealed a distinct separation between the control group (CK), the clonazepam group, and the serotonin + clonazepam group, indicating significant alterations in the metabolic profile following clonazepam and serotonin + clonazepam treatments. In contrast, the changes in the metabolic profile after the serotonin treatment were relatively minor compared with those induced by clonazepam (Figure 4).

### 3.4. Partial Least Squares Regression Model

**Figure 5:**
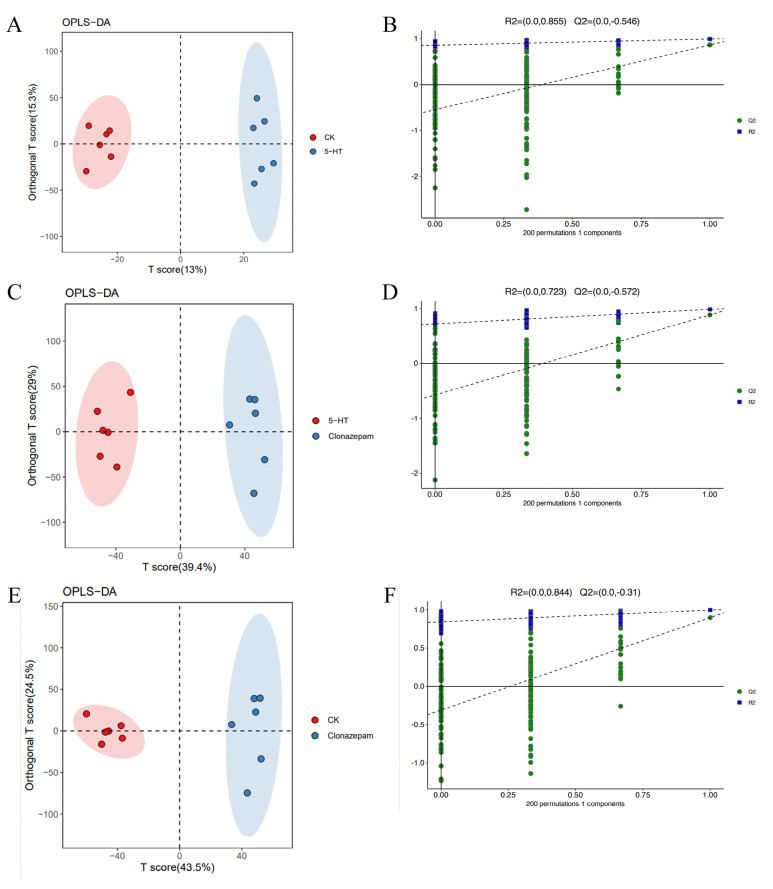
Comparison between groups under three treatments of *P. trituberculatus* larvae. (**A**,**C**,**E**) OPLS-DA diagrams of comparisons among the control group, the serotonin group, and the serotonin + clonazepam group. (**B**,**D**,**F**) Substitution test plots of three group comparisons.

PCA is an unsupervised discriminant analysis technique, but it is unable to categorize samples and eliminate intra-group errors. Consequently, outliers within groups can substantially influence the outcome, often resulting in principal components that are skewed toward these outliers. Therefore, a supervised orthogonal projections to latent structures discriminant analysis (OPLS-DA) was employed in this study. OPLS-DA effectively eliminates irrelevant variation within data while incorporating group information and identifying variables that contribute most significantly to discrimination. Figure 5 shows the OPLS-DA score plots. The OPLS-DA results showed that each group was distinctly positioned in the left or right quadrants, indicating clear differentiation. There were significant differences in the metabolites among the three groups. OPLS-DA provided a more refined analysis. Finally, to ensure the reliability of the experimental results and to check for overfitting, we conducted a permutation test using 200 permutations. The validity and stability of the model were ensured by establishing the OPLS-DA model and calculating the R2 and Q2 values. R2 and Q2 are the intercept values with the y-axis after linear regression of R2Y and Q2Y on the original model. As shown in Figure 5, the Q2 regression lines of the control and serotonin groups, the serotonin and clonazepam groups, and the control and clonazepam groups all intersected below zero on the vertical axis, indicating that the model was effective and stable.

### 3.5. Screening for Differential Metabolites in P. trituberculatus in the CK, Serotonin, and Clonazepam Groups

**Figure 6:**
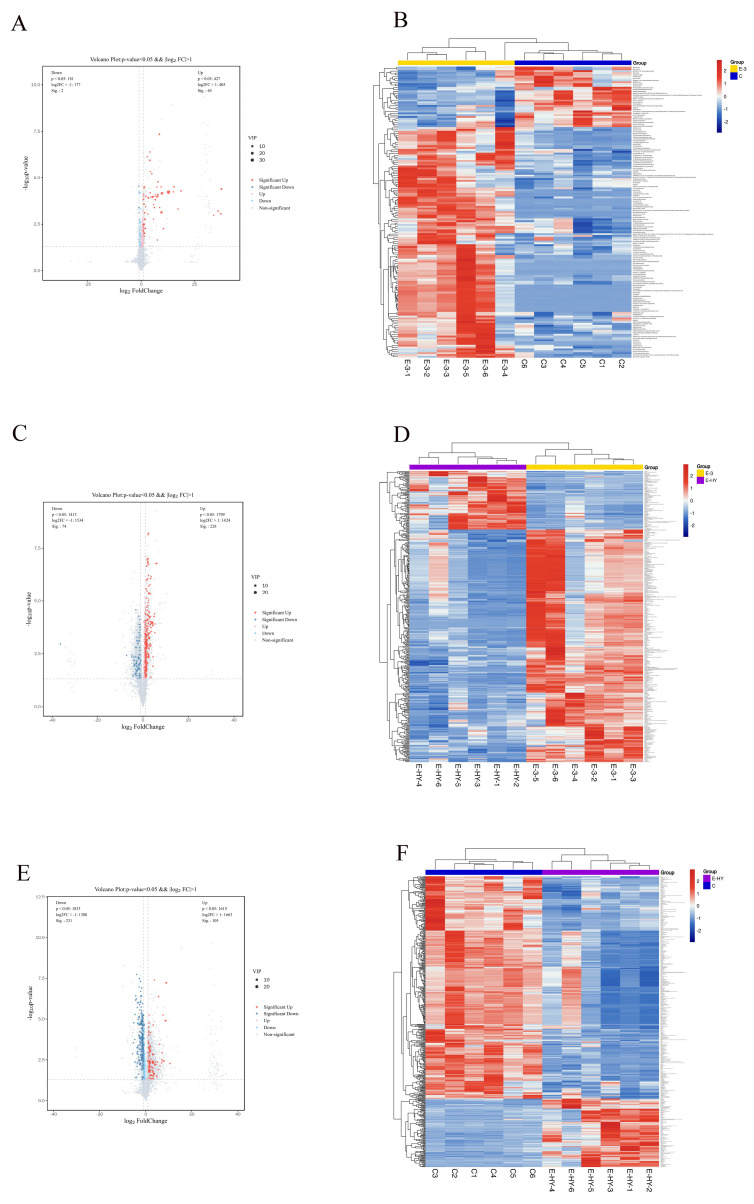
Visual analysis of differential metabolites. (**A**,**C**,**E**) Volcano plots: visual presentation of differential metabolites between three different treatment groups. (**B**,**D**,**F**) Heat maps: clustering analysis of the three treatment groups.

A total of 5515 metabolites were detected using untargeted metabolomics analysis. Differential metabolites were selected based on the criteria of variable importance in projection (VIP) values (VIP ≥ 1) and *p*-values (*p* < 0.05) derived from the OPLS-DA. Intergroup comparisons were conducted among the control group (CK), the serotonin group, and the clonazepam group. Within the CK and serotonin groups, 67 differential metabolites were identified, of which 65 were upregulated and two were downregulated, primarily comprising lipids and lipid-like molecules, organic acids and their derivatives, phenyls and phenylpropanoids, and polyketides. In the comparison between the 5-HT and clonazepam groups, 302 differential metabolites were identified, predominantly alkaloids and their derivatives, phenylpropanoids and polyketides, phenyls, lipids and lipid-like molecules, and organic acids and their derivatives, with 228 upregulated and 74 downregulated metabolites. Between the CK and clonazepam groups, 336 differential metabolites were identified, with 105 upregulated and 231 downregulated metabolites, consisting of alkaloids and their derivatives, nucleosides, nucleotides and their analogs, phenyls, lipids and lipid-like molecules, and organic acids and their derivatives. Volcano plots were employed to visually represent the changes in metabolites (Figure 6), with red dots indicating upregulated metabolites and blue dots indicating downregulated metabolites. Finally, cluster heat map analysis was performed on the differential metabolites to intuitively display the variation among samples and the differences in metabolite expression across different samples.

### 3.6. Metabolic Pathway Enrichment Analysis for Differential Metabolites

**Figure 7:**
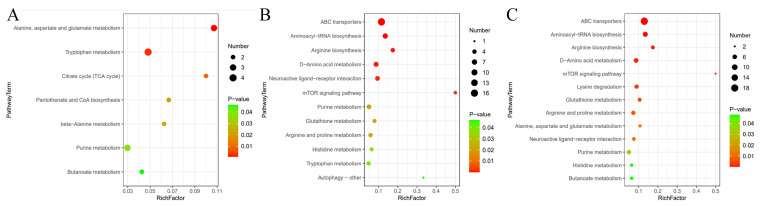
Analysis of different metabolic pathways between the control and serotonin groups, the serotonin and serotonin + clonazepam groups, and the control and serotonin + clonazepam groups. (**A**) The control and serotonin groups. (**B**) The serotonin and serotonin + clonazepam groups. (**C**) The control and serotonin + clonazepam groups.

Further KEGG pathway analysis and enrichment analysis of the above-mentioned differential metabolites revealed significant differential pathways between the three groups, and bubble charts were constructed to illustrate these findings (Figure 7). Notably, pathways more significantly affected the serotonin group compared with the control group, including alanine, aspartate and glutamate metabolism, tryptophan metabolism, the citrate cycle, pantothenate and CoA biosynthesis, beta-alanine metabolism, purine metabolism, and butanoate metabolism. The primary pathways in the serotonin group compared with the clonazepam group included ABC transporters, aminoacyl-tRNA biosynthesis, arginine biosynthesis, d-amino acid metabolism, neuroactive ligand–receptor interactions, the mTOR signaling pathway, purine metabolism, glutathione metabolism, arginine and proline metabolism, histidine metabolism, tryptophan metabolism, and autophagy—other. Finally, significant metabolic pathways identified between the CK and clonazepam groups included ABC transporters, aminoacyl-tRNA biosynthesis, arginine biosynthesis, D-amino acid metabolism, the mTOR signaling pathway, lysine degradation, glutathione metabolism, arginine and proline metabolism, alanine, aspartate and glutamate metabolism, neuroactive ligand–receptor interaction, purine metabolism, histidine metabolism, and butanoate metabolism. The analysis indicated that treatment with serotonin influenced 38 metabolic pathways, with 7 of these pathways showing significant dysregulation. An investigation of the differential metabolites enriched in these altered pathways revealed that tryptophan metabolism, which exhibited a notable upregulation of serotonin, may be associated with anxiety. However, a significant difference in tryptophan metabolism persisted between the serotonin and clonazepam groups, with this difference transitioning from upregulation to a downregulation, aligning the pathway’s activity to levels observed in the control group. In the comparison between the CK and clonazepam groups, there was no difference in tryptophan metabolism. This suggests that the effect of serotonin on tryptophan metabolism could be mitigated by clonazepam.

## 4. Discussion

Serotonin is a chemical neurotransmitter widely present in humans and animals, regulating various physiological processes and mental functions. In rodent studies, serotonin has been demonstrated to regulate anxiety through various pathways, including binding to corresponding receptors and affecting neuronal growth and plasticity [56]. Notably, anxiety research has primarily focused on rodents and other species of mammals. Due to the lack of complex neural structures in crustaceans, related studies have been relatively scarce. Recent research revealed that *Procambarus clarkii*, when stressed, exhibited anxiety-like behaviors in a cross-maze test similar to those of rodents, and this behavior could be regulated by serotonin, confirming the possibility of anxiety-like behaviors in invertebrates [35]. This discovery indicated that such anxiety-like behaviors were not exclusive to vertebrates and that research into crustacean anxiety behaviors was feasible. In our study, we demonstrated that exogenous serotonin led to an increase in anxiety-like behaviors in the zoea of *P. trituberculatus* in an OFT-like, as reflected by the significantly reduced time spent by the zoea in the central area of the open field-like test. To reveal these anxiety-like behaviors, we treated the zoea with the benzodiazepine drug clonazepam and observed their changes in behavior. Clonazepam regulates gamma-aminobutyric acid-A receptors, opens chloride ion channels, and hyperpolarizes neurons, thereby reducing neuronal excitability and alleviating anxiety [57,58,59]. The results showed that anxiety-like behaviors could be eliminated by clonazepam, consistent with studies in zebrafish [60]. This finding reconfirmed the presence of anxiety-like behaviors in invertebrates, indicating that, despite lacking complex neural structures, crustaceans still exhibit anxiety-like behaviors and thus provide a simpler nervous system model for anxiety research and help scientists with a better understanding of the basic mechanism of anxiety.

Anxiety is a common disorder, yet there is limited research on the levels of metabolites under anxiety conditions. Metabolomics, by studying the dynamic changes in small molecular metabolites, can reveal the physiological states of organisms during life activities [61]. Current research using metabolomics has revealed alterations in various key pathways in rodents and mammals under conditions of anxiety. These include changes in energy metabolism induced by a high-fat diet [62], changes in amino acid metabolism related to arginine and proline metabolism, and alterations in amino acids, such as alanine, aspartate, and glutamate [63]. It is noteworthy that factors affecting this metabolic pathway are commonly encountered in daily life. Heavy metal toxicity, such as that from mercury, lead, and cadmium, can lead to abnormal behaviors in aquatic animals, including reduced activity, increased avoidance behavior, and other negative responses, which have been linked to anxiety. Some scholars’ research has confirmed that heavy metal poisoning disrupts both the physiological state and behavior of aquatic animals [64]. Metabolic changes in neurotransmitters, including serotonin, dopamine, and gamma-aminobutyric acid, have also been identified [65]. Additionally, alterations in lipid metabolism and oxidative stress pathways induced by purines and indole derivatives, such as xanthine and indoxyl sulfate, have been observed [66]. However, metabolomic research on anxiety behavior in crustaceans is still in its infancy. To further understand the underlying complex biological mechanisms, we used metabolomics analysis to reveal the differential changes in metabolites of zoea *P. trituberculatus* under anxiety conditions. The results showed that 67 differential metabolites were identified between the control and serotonin groups. Metabolic pathway enrichment analysis revealed that a pathway related to anxiety was tryptophan metabolism, which included significant differential changes in four metabolites: indole-3-pyruvate, serotonin, N-acetyl serotonin, and L-kynurenine. Tryptophan metabolism is one of the key pathways affecting anxiety. The serotonin pathway [67] and tryptophan–kynurenine metabolism [68] are important metabolic pathways in tryptophan metabolism, with inflammation [69] and other pathways also controlling the production of anxiety, to some extent. The serotonin pathway involves the conversion of tryptophan to serotonin regulated by tryptophan hydroxylase, followed by the production of serotonin by amino acid decarboxylase. The changes in serotonin levels influence the occurrence of anxiety [70]. Tryptophan–kynurenine metabolism can convert tryptophan into kynurenine through tryptophan 2,3-dioxygenase or indoleamine 2,3-dioxygenase [71], eventually forming neurologically relevant metabolites, such as 3-hydroxykynurenine, quinolinic acid, niacin, and nicotinamide adenine dinucleotide [72], which regulate anxiety through neural control.

N-acetyl serotonin is a significant factor in the biosynthesis of serotonin and melatonin. Studies have shown that, as a precursor to melatonin, high concentrations of N-acetyl serotonin can inhibit anxious behavior in mice. The anxiolytic effect of n-Ac-serotonin is achieved through melatonin or the activation of TrkB receptors [73,74]. As an invertebrate, the pineal gland structure in crustaceans differs from that of vertebrates, but is still capable of melatonin synthesis. In this study, there was a significant elevation in N-acetyl serotonin levels, but this did not influence melatonin content or exhibit an anxiolytic effect. The elevation of N-acetyl serotonin may be the result of a sudden surge in serotonin levels. In addition, whether the TrkB receptor exists in invertebrates remains unclear.

L-kynurenine is an intermediate product in the tryptophan metabolic pathway produced by the conversion of tryptophan by tryptophan 2,3-dioxygenase and indoleamine 2,3-dioxygenase [75]. Indoleamine 2,3-dioxygenase has been demonstrated to mediate peripheral immune attacks by lipopolysaccharides, subsequently inducing anxious behaviors in mice. Mice treated with L-kynurenine also exhibit anxiety-like behaviors [76,77]. Moreover, a significant increase in kynurenine levels has been detected in mice with inflammation-induced anxiety [78]. Currently, the role of kynurenine in anxiety involves multiple pathways, including neurotransmitters [79], inflammation [78], and neuroprotection [80]. In this study, the content of L-kynurenine was significantly increased. Through annotation analysis of differential metabolite pathways, the result showed a significant elevation in the level of tryptophan 2,3-dioxygenase, indicating its involvement in anxiety regulation in *P. trituberculatus*. This may be associated with the regulation of tryptophan metabolism subsequently influencing neurotransmitter synthesis. The serotonin levels of the crab zoea were significantly increased in this study.

We identified metabolites potentially related to anxiety, such as folic acid. Folic acid, a vitamin, is used in prenatal care in appropriate amounts to reduce the likelihood of depressive behaviors in offspring [81]. Studies have shown that abnormal DNA methylation can lead to depressive behaviors [82]. Supplementing with folic acid helps to maintain normal DNA methylation, ensuring the healthy development of offspring [83]. Furthermore, folic acid can improve anxiety-like behaviors by regulating neurotransmitter levels [84], oxidative stress [85], and homocysteine levels [86]. In *P. trituberculatus*, it is plausible that reduced levels of folic acid may have led to behavioral changes, indirectly affecting the onset of anxiety.

In anxiety studies, researchers often select mammals such as rats as experimental subjects to measure anxiety states and explore the underlying biological mechanisms [87]. Mammals share high similarity with humans in genetics, behavioral characteristics, and the nervous system, providing valuable models for the study of related human diseases. For example, in the study of anxious behavior, extensive research data have demonstrated that mammals exhibit behaviors such as avoidance and fear when encountering threats [88,89]. These behaviors are observable and quantifiable, and analyzing them alongside the underlying biological mechanisms can offer effective references and suggestions for human anxiety research. Invertebrates also display defense or escape behaviors when threatened, but these are more stress responses driven by instinct [90]. Even though neurotransmitters and hormones such as serotonin and dopamine are present, they primarily function in stress responses. Whether invertebrates truly experience anxiety remains a matter of debate. Nonetheless, research on anxiety-like behaviors in invertebrates has made progress [91]. Researchers have discovered that, based on the phototaxis of Gammarus, a light–dark cross maze successfully yielded results indicative of anxiety-like behavior in these creatures. This finding suggests that invertebrates can exhibit anxiety-like behaviors. However, due to the limited research on the neurobiological mechanisms of invertebrates, their anxiety-like behaviors can only be studied and analyzed from behavioral and physiological perspectives, rather than actual emotional experiences. Whether invertebrates genuinely possess such emotions requires more in-depth research.

## 5. Conclusions

This study has revealed the regulatory role of serotonin in anxiety-like behaviors of zoea in swimming crab, identifying changes in differential metabolites and metabolic pathways induced by serotonin. These findings confirm the possibility of anxiety-like behaviors in invertebrates. Despite their less complex nervous system than that of mammals, the results align with previous discoveries in crustaceans. This study lays the groundwork for anxiety research in invertebrates, indicating that future studies in this area are both feasible and meaningful.

## Data Availability

Data will be made available on request. The data are not publicly available due to privacy.

## References

[1] Khakpay R., Khakpai F. (2020). Modulation of anxiety behavior in gonadectomized animals. Acta Neurobiol. Exp..

[2] Heisler L.K., Zhou L., Bajwa P., Hsu J., Tecott L.H. (2007). Serotonin 5-HT_2C_ receptors regulate anxiety-like behavior. Genes Brain Behav..

[3] Malan-Muller S., Valles-Colomer M., Palomo T., Leza J.C. (2023). The gut-microbiota-brain axis in a Spanish population in the aftermath of the COVID-19 pandemic: Microbiota composition linked to anxiety, trauma, and depression profiles. Gut Microbes.

[4] Choueiry N., Salamoun T., Jabbour H., El Osta N., Hajj A., Rabbaa Khabbaz L. (2016). Insomnia and relationship with anxiety in university students: A cross-sectional designed study. PLoS ONE.

[5] Du M., Peng Y., Li Y., Zhu Y., Yang S., Li J., Zou F., Wang Y., Wu X., Zhang Y. (2022). Effect of trait anxiety on cognitive flexibility: Evidence from event-related potentials and resting-state EEG. Biol. Psychol..

[6] Lutz J., Mashal N., Kramer A., Suresh M., Gould C., Jordan J.T., Wetherell J.L., Beaudreau S.A. (2020). A case report of problem solving therapy for reducing suicide risk in older adults with anxiety disorders. Clin. Gerontol..

[7] Kobayashi H., Martinez de Toda I., Sanz-San Miguel L., De la Fuente M. (2021). Sex-related differences in behavioural markers in adult mice for the prediction of lifespan. Biogerontology.

[8] Rangassamy M., Dalmas M., Féron C., Gouat P., Rödel H.G. (2015). Similarity of personalities speeds up reproduction in pairs of a monogamous rodent. Anim. Behav..

[9] Meyer N., Richter S.H., Schreiber R.S., Kloke V., Kaiser S., Lesch K.P., Sachser N. (2016). The unexpected effects of beneficial and adverse social experiences during adolescence on anxiety and aggression and their modulation by genotype. Front. Behav. Neurosci..

[10] Patki G., Atrooz F., Alkadhi I., Solanki N., Salim S. (2015). High aggression in rats is associated with elevated stress, anxiety-like behavior, and altered catecholamine content in the brain. Neurosci. Lett..

[11] Heinz D.E., Schottle V.A., Nemcova P., Binder F.P., Ebert T., Domschke K., Wotjak C.T. (2021). Exploratory drive, fear, and anxiety are dissociable and independent components in foraging mice. Transl. Psychiatry.

[12] Mohammad F., Aryal S., Ho J., Stewart J.C., Norman N.A., Tan T.L., Eisaka A., Claridge-Chang A. (2016). Ancient anxiety pathways influence Drosophila defense behaviors. Curr. Biol..

[13] Kompagne H., Bardos G., Szenasi G., Gacsalyi I., Harsing L.G., Levay G. (2008). Chronic mild stress generates clear depressive but ambiguous anxiety-like behaviour in rats. Behav. Brain Res..

[14] Ohl F., Roedel A., Binder E., Holsboer F. (2003). Impact of high and low anxiety on cognitive performance in a modified hole board test in C57BL/6 and DBA/2 mice. Eur. J. Neurosci..

[15] Sparling J.E., Baker S.L., Bielajew C. (2018). Effects of combined pre- and post-natal enrichment on anxiety-like, social, and cognitive behaviours in juvenile and adult rat offspring. Behav. Brain Res..

[16] Lalonde R., Strazielle C. (2008). Relations between open-field, elevated plus-maze, and emergence tests as displayed by C57/BL6J and BALB/c mice. J. Neurosci. Methods.

[17] Hope B.V., Hamilton T.J., Hurd P.L. (2019). Submerged plus maze: A novel test for studying anxiety-like behaviour in fish. Behav. Brain Res..

[18] Lim L.W., Temel Y., Visser-Vandewalle V., Steinbusch H., Schruers K., Hameleers R., Esquivel G., Griez E., Blokland A. (2008). Effect of buspirone on the behavioral regulation of rats in low versus high anxiety conditions. Arzneimittelforschung.

[19] Maldonado E., Navarro J.F. (2000). Effects of 3,4-methylenedioxy-methamphetamine (MDMA) on anxiety in mice tested in the light-dark box. Prog. Neuropsychopharmacol. Biol. Psychiatry.

[20] Li X.L., Aou S., Hori T., Oomura Y. (2002). Spatial memory deficit and emotional abnormality in OLETF rats. Physiol. Behav..

[21] Lu T., Shen Y., Cui G.X., Yin F.W., Yu Z.L., Zhou D.Y. (2020). Detailed analysis of lipids in edible viscera and muscles of cooked crabs *Portunus trituberculatus* and *Portunus pelagicus*. J. Aquat. Food Prod. Technol..

[22] He J., Wan L., Yu H., Peng Y., Zhang D., Xu W. (2022). Effect of water temperature on embryonic development of *Protunus trituberculatus* in an off-season breeding mode. Front. Mar. Sci..

[23] Liu B., Zhang X., Wang Z., Li W., Zhang Q., Liu Q., Liu W., Zhang L., Liu Y., Wang C. (2021). Genetic pattern fluctuations in wild swimming crab populations, under the influence of continuous mass stock enhancement. Fish. Res..

[24] Chen J., Chen X., Mu C., Wang C., Ye Y., Li R., Song W., Shi C., Liu L., Wang H. (2023). Comparative transcriptome analysis reveals the growth and development in larval stages of the swimming crab *Portunus trituberculatus*. Front. Mar. Sci..

[25] Liang Q., Zhu B., Liu D., Lu Y., Zhang H., Wang F. (2023). Serotonin and dopamine regulate the aggressiveness of swimming crabs (*Portunus trituberculatus*) in different ways. Physiol. Behav..

[26] Liu D., Wang F., Yang C., Hu N., Sun Y. (2017). Starvation and a conspecific competitor influence multiple predator effects in a swimming crab (*Portunus trituberculatus*)-Manila clam (*Ruditapes philippinarum*) foraging system. J. Exp. Mar. Biol. Ecol..

[27] Liu D., Wang F., Lu Y., Zhu B., Zhang H. (2022). Effects of stocking density on a typical crab-clam polyculture system: Behavioral mechanisms of predation and competition in swimming crab (*Portunus trituberculatus*). Aquaculture.

[28] Zhu B., Zhang H., Lu Y., Wang F., Liu D. (2022). The effect of intruder density on territoriality and dominance in male swimming crab (*Portunus trituberculatus*). Animals.

[29] Kudryavtseva N.N., Bondar N.P., Avgustinovich D.F. (2002). Association between experience of aggression and anxiety in male mice. Behav. Brain Res..

[30] Podhorna J., Krsiak M. (2000). Behavioural effects of a benzodiazepine receptor partial agonist, Ro 19-8022, in the social conflict test in mice. Behav. Pharmacol..

[31] Yu W.S., Guan L., Kai Tan S.Z., Shrestha S., Or Y.Z., Lufkin T., Lin V.C., Lim L.W. (2022). Tetratricopeptide repeat domain 9A knockout induces social anxiety and impairs offense behaviors in female mice. Iran. J. Basic Med. Sci..

[32] Fossat P., Bacque-Cazenave J., De Deurwaerdere P., Delbecque J.P., Cattaert D. (2014). Comparative behavior. Anxiety-like behavior in crayfish is controlled by serotonin. Science.

[33] Xu P., He Y., Cao X., Valencia-Torres L., Yan X., Saito K., Wang C., Yang Y., Hinton A., Zhu L. (2017). Activation of serotonin 2C receptors in dopamine neurons inhibits binge-like eating in mice. Biol. Psychiatry.

[34] Li X., He C., Shen M., Wang M., Zhou J., Chen D., Zhang T., Pu Y. (2024). Effects of aqueous extracts and volatile oils prepared from Huaxiang Anshen decoction on p-chlorophenylalanine-induced insomnia mice. J. Ethnopharmacol..

[35] Lin D., Parsons L.H. (2002). Anxiogenic-like effect of serotonin_1B_ receptor stimulation in the rat elevated plus-maze. Pharmacol. Biochem. Behav..

[36] Yu W., Yen Y.C., Lee Y.H., Tan S., Xiao Y., Lokman H., Ting A.K.T., Ganegala H., Kwon T., Ho W.K. (2019). Prenatal selective serotonin reuptake inhibitor (SSRI) exposure induces working memory and social recognition deficits by disrupting inhibitory synaptic networks in male mice. Mol. Brain.

[37] Bano S., Sharif H., Sajid F., Hamid S.B., Badawy A.A. (2023). Liver tryptophan 2,3-dioxygenase: A determinant of anxiety-like behaviour—Studies with chronic nicotine administration in rats. Behav. Pharmacol..

[38] Ma M., Quan H., Chen S., Fu X., Zang L., Dong L. (2023). The anxiolytic effect of polysaccharides from stellariae radix through monoamine neurotransmitters, HPA axis, and ECS/ERK/CREB/BDNF signaling pathway in stress-induced male rats. Brain Res. Bull..

[39] Tao Y., Zhou H., Li Z., Wu H., Wu F., Miao Z., Shi H., Huang F., Wu X. (2024). TGR5 deficiency-induced anxiety and depression-like behaviors: The role of gut microbiota dysbiosis. J. Affect. Disord..

[40] Naslund J., Studer E., Johansson E., Eriksson E. (2016). Effects of gonadectomy and serotonin depletion on inter-individual differences in anxiety-like behaviour in male Wistar rats. Behav. Brain Res..

[41] Echeverri N., Govendir M. (2022). Does the selective serotonin reuptake inhibitor (SSRI) fluoxetine modify canine anxiety related behaviour?. Vet. Evid..

[42] Glover M.E., Clinton S.M. (2016). Of rodents and humans: A comparative review of the neurobehavioral effects of early life SSRI exposure in preclinical and clinical research. Int. J. Dev. Neurosci..

[43] Stahl S.M. (1998). Mechanism of action of serotonin selective reuptake inhibitors: Serotonin receptors and pathways mediate therapeutic effects and side effects. J. Affect. Disord..

[44] Willadsen M., Schwarting R.K.W., Wohr M. (2023). Acute anxiogenic effects of escitalopram are associated with mild alterations in D-amphetamine-induced behavior and social approach evoked by playback of 50-kHz ultrasonic vocalizations in rats. Neuropharmacology.

[45] Martin J.M., Bertram M.G., Saaristo M., Fursdon J.B., Hannington S.L., Brooks B.W., Burket S.R., Mole R.A., Deal N.D.S., Wong B.B.M. (2019). Antidepressants in surface waters: Fluoxetine influences mosquitofish anxiety-related behavior at environmentally relevant levels. Environ. Sci. Technol..

[46] Kim S., Kim J., Yun E.J., Kim K.H. (2016). Food metabolomics: From farm to human. Curr. Opin. Biotechnol..

[47] Tran H., McConville M., Loukopoulos P. (2020). Metabolomics in the study of spontaneous animal diseases. J. Vet. Diagn. Investig..

[48] Wu W., Zhang L., Zheng X., Huang Q., Farag M.A., Zhu R., Zhao C. (2022). Emerging applications of metabolomics in food science and future trends. Food Chem. X.

[49] McGee E.E., Kiblawi R., Playdon M.C., Eliassen A.H. (2019). Nutritional metabolomics in cancer epidemiology: Current trends, challenges, and future directions. Curr. Nutr. Rep..

[50] Qin N., Qin M., Shi W., Kong L., Wang L., Xu G., Guo Y., Zhang J., Ma Q. (2022). Investigation of pathogenesis of hyperuricemia based on untargeted and targeted metabolomics. Sci. Rep..

[51] Fukusaki E., Kobayashi A. (2005). Plant metabolomics: Potential for practical operation. J. Biosci. Bioeng..

[52] Ramirez T., Daneshian M., Kamp H., Bois F.Y., Clench M.R., Coen M., Donley B., Fischer S.M., Ekman D.R., Fabian E. (2013). Metabolomics in toxicology and preclinical research. ALTEX.

[53] Filiou M.D., Asara J.M., Nussbaumer M., Teplytska L., Landgraf R., Turck C.W. (2014). Behavioral extremes of trait anxiety in mice are characterized by distinct metabolic profiles. J. Psychiatr. Res..

[54] Zhang Y., Filiou M.D., Reckow S., Gormanns P., Maccarrone G., Kessler M.S., Frank E., Hambsch B., Holsboer F., Landgraf R. (2011). Proteomic and metabolomic profiling of a trait anxiety mouse model implicate affected pathways. Mol. Cell. Proteom..

[55] Liu Y., Zhao W., Lu Y., Zhao Y., Zhang Y., Dai M., Hai S., Ge N., Zhang S., Huang M. (2022). Systematic metabolic characterization of mental disorders reveals age-related metabolic disturbances as potential risk factors for depression in older adults. MedComm.

[56] Piszczek L., Schlax K., Wyrzykowska A., Piszczek A., Audero E., Thilo Gross C. (2013). Serotonin 1A auto-receptors are not sufficient to modulate anxiety in mice. Eur. J. Neurosci..

[57] Louiset E., Valentijn J.A., Vaudry H., Cazin L. (1992). Central-type benzodiazepines modulate GABAA receptor chloride channels in cultured pituitary melanotrophs. Mol. Brain Res..

[58] Ochoa J.G., Kilgo W.A. (2016). The role of benzodiazepines in the treatment of epilepsy. Curr. Treat. Options Neurol..

[59] Yang Y., Wang B., Zhong Z., Chen H., Ding W., Hoi M.P.M. (2021). Clonazepam attenuates neurobehavioral abnormalities in offspring exposed to maternal immune activation by enhancing GABAergic neurotransmission. Biochem. Pharmacol..

[60] Gusso D., Altenhofen S., Fritsch P.M., Rubensam G., Bonan C.D. (2021). Oxytetracycline induces anxiety-like behavior in adult zebrafish. Toxicol. Appl. Pharmacol..

[61] Li G., Jian T., Liu X., Lv Q., Zhang G., Ling J. (2022). Application of metabolomics in fungal research. Molecules.

[62] Demianchuk O., Vatashchuk M., Gospodaryov D., Hurza V., Ivanochko M., Derkachov V., Berezovskyi V., Lushchak O., Storey K.B., Bayliak M. (2024). High-fat high-fructose diet and alpha-ketoglutarate affect mouse behavior that is accompanied by changes in oxidative stress response and energy metabolism in the cerebral cortex. Biochim. Biophys. Acta Gen. Subj..

[63] Yin X., Deng Y., Guo C., Ding C., Xu J., Wu F. (2023). Behavioral changes and metabolic responses of adult zebrafish (Danio Rerio) exposed to methamphetamine. ACS ES&T Water.

[64] Lettieri G., Marinaro C., Notariale R., Perrone P., Lombardi M., Trotta A., Troisi J., Piscopo M. (2023). Impact of Heavy Metal Exposure on *Mytilus galloprovincialis* Spermatozoa: A Metabolomic Investigation. Metabolites.

[65] Custodio R.J.P., Hobloss Z., Myllys M., Hassan R., Gonzalez D., Reinders J., Bornhorst J., Weishaupt A.K., Seddek A.L., Abbas T. (2023). Cognitive functions, neurotransmitter alterations, and hippocampal microstructural changes in mice caused by feeding on western diet. Cells.

[66] Puurunen J., Tiira K., Lehtonen M., Hanhineva K., Lohi H. (2016). Non-targeted metabolite profiling reveals changes in oxidative stress, tryptophan and lipid metabolisms in fearful dogs. Behav. Brain Funct..

[67] Fang Y., Li Y., Liao X., Deng J., Wang Q., Liang J., Yan B. (2023). *Corydalis yanhusuo* polysaccharides ameliorate chronic stress-induced depression in mice through gut microbiota-derived short-chain fatty acid activation of 5-hydroxytryptamine signaling. J. Med. Food.

[68] Ye F., Dong M.C., Xu C.X., Jiang N., Chang Q., Liu X.M., Pan R.L. (2023). Effects of different chronic restraint stress periods on anxiety- and depression-like behaviors and tryptophan-kynurenine metabolism along the brain-gut axis in C57BL/6N mice. Eur. J. Pharmacol..

[69] Alizadeh Pahlavani H. (2024). Possible role of exercise therapy on depression: Effector neurotransmitters as key players. Behav. Brain Res..

[70] Gao K., Farzi A., Ke X., Yu Y., Chen C., Chen S., Yu T., Wang H., Li Y. (2022). Oral administration of *Lactococcus lactis* WHH2078 alleviates depressive and anxiety symptoms in mice with induced chronic stress. Food Funct..

[71] Badawy A.A. (2023). The kynurenine pathway of tryptophan metabolism: A neglected therapeutic target of COVID-19 pathophysiology and immunotherapy. Biosci. Rep..

[72] Zhou M., Fan Y., Xu L., Yu Z., Wang S., Xu H., Zhang J., Zhang L., Liu W., Wu L. (2023). Microbiome and tryptophan metabolomics analysis in adolescent depression: Roles of the gut microbiota in the regulation of tryptophan-derived neurotransmitters and behaviors in human and mice. Microbiome.

[73] Jang S.W., Liu X., Pradoldej S., Tosini G., Chang Q., Iuvone P.M., Ye K. (2010). N-acetylserotonin activates TrkB receptor in a circadian rhythm. Proc. Natl. Acad. Sci. USA.

[74] Kopp C., Vogel E., Rettori M., Delagrange P., Misslin R. (2000). Anxiolytic-like properties of melatonin receptor agonists in mice: Involvement of mt1 and/or MT2 receptors in the regulation of emotional responsiveness. Neuropharmacology.

[75] Orabona C., Puccetti P., Vacca C., Bicciato S., Luchini A., Fallarino F., Bianchi R., Velardi E., Perruccio K., Velardi A. (2006). Toward the identification of a tolerogenic signature in IDO-competent dendritic cells. Blood.

[76] O’Connor J.C., Lawson M.A., Andre C., Moreau M., Lestage J., Castanon N., Kelley K.W., Dantzer R. (2009). Lipopolysaccharide-induced depressive-like behavior is mediated by indoleamine 2,3-dioxygenase activation in mice. Mol. Psychiatry.

[77] Salazar A., Gonzalez-Rivera B.L., Redus L., Parrott J.M., O’Connor J.C. (2012). Indoleamine 2,3-dioxygenase mediates anhedonia and anxiety-like behaviors caused by peripheral lipopolysaccharide immune challenge. Horm. Behav..

[78] Bercik P., Verdu E.F., Foster J.A., Macri J., Potter M., Huang X., Malinowski P., Jackson W., Blennerhassett P., Neufeld K.A. (2010). Chronic gastrointestinal inflammation induces anxiety-like behavior and alters central nervous system biochemistry in mice. Gastroenterology.

[79] Ibos K.E., Bodnar E., Dinh H., Kis M., Marvanykovi F., Kovacs Z.Z.A., Siska A., Foldesi I., Galla Z., Monostori P. (2023). Chronic kidney disease may evoke anxiety by altering CRH expression in the amygdala and tryptophan metabolism in rats. Pflug. Arch..

[80] Varga D., Heredi J., Kanvasi Z., Ruszka M., Kis Z., Ono E., Iwamori N., Iwamori T., Takakuwa H., Vecsei L. (2015). Systemic L-Kynurenine sulfate administration disrupts object recognition memory, alters open field behavior and decreases c-Fos immunopositivity in C57Bl/6 mice. Front. Behav. Neurosci..

[81] Ono T., Hino K., Kimura T., Uchimura Y., Ashihara T., Higa T., Kojima H., Murakami T., Udagawa J. (2022). Excessive folic acid intake combined with undernutrition during gestation alters offspring behavior and brain monoamine profiles. Congenit. Anom..

[82] Sahara Y., Matsuzawa D., Ishii D., Fuchida T., Goto T., Sutoh C., Shimizu E. (2019). Paternal methyl donor deficient diets during development affect male offspring behavior and memory-related gene expression in mice. Dev. Psychobiol..

[83] Tuo L.J., Song X.Y., Zhu Y.Y., He H.N., Song Y.P., Chen D.Z., Zheng X.M., Zhang H., Xu D.X. (2023). Gestational folic acid supplement prevents vitamin D deficiency-induced depression-like behavior by reversing cortical DNA hypomethylation in adult offspring. J. Steroid Biochem. Mol. Biol..

[84] Onaolapo O.J., Olofinnade A.T., Ojo F.O., Falade J., Onaolapo A.Y. (2023). Prepubertal continuous dietary folate fortification enhances the brain function of adult mice by modulating antioxidant status, inflammation, and brain neurotransmitter levels. Antinflammatory Antiallergy Agents Med. Chem..

[85] Hosseini S.H., Khabbazhosseini Z.S., Khatibi S., Yahosseini A., Borhaninejad N., Beheshti F., Kakhki S. (2023). Folic acid supplementation improved nicotine withdrawal-induced of memory loss via affecting oxidative status, inflammatory response, cholinergic activity, BDNF and amyloid-B in adolescent male rat. Neurosci. Lett..

[86] Shemirani F., Titcomb T.J., Saxby S.M., Eyck P.T., Rubenstein L.M., Hoth K.F., Snetselaar L.G., Wahls T.L. (2023). Association of serum homocysteine, folate, and vitamin B_12_ and mood following the Swank and Wahls elimination dietary interventions in relapsing-remitting multiple sclerosis: Secondary analysis of the WAVES trial. Mult. Scler. Relat. Disord..

[87] Bult C.J., Blake J.A., Smith C.L., Kadin J.A., Richardson J.E. (2019). Mouse Genome Database (MGD) 2019. Nucleic Acids Res..

[88] Perkins R., Boal C., Rollins D., Perez R.M. (2014). Northern bobwhite predator avoidance behavior in response to varying types of threat. J. Wildl. Manag..

[89] Quah S.K.L., Cockcroft G.J., McIver L., Santangelo A.M., Roberts A.C. (2020). Avoidant coping style to high imminence threat is linked to higher anxiety-like behavior. Front. Behav. Neurosci..

[90] Paçal E., Gümüş A.B., Günal A.Ç., Erkmen B., Arslan P., Yıldırım Z., Erkoç F. (2022). Oxidative stress response as biomarker of exposure of a freshwater invertebrate model organism (Unio mancus Lamarck, 1819) to antifouling copper pyrithione. Pestic. Phytomedicine/Pestic. Fitomedicina.

[91] Greisberg J., Gorroochurn P., Greisberg J.K. (2022). Sustained anxiety-like behavior in crayfish exposed to thermal burn. J. Stud. Res..

